# Supplementary material for: Development of the mandibular curve of spee and maxillary compensating curve: A finite element model
Source: PLoS One. 2019 Dec 26;14(12):e0221137. doi: 10.1371/journal.pone.0221137 (PMC6932755; doi:10.1371/journal.pone.0221137)
Supplement: S1 Video — https://www.dentistry.uiowa.edu/orthodontics-curveofspeeshortradius-yt.html. (DOCX) [file pone.0221137.s001.docx]

**S1 Video. World Wide Web address of the FEM simulation of human masticatory movements seen in the sagittal plane for C_ROT_ = 100 mm.** <https://www.dentistry.uiowa.edu/orthodontics-curveofspeeshortradius-yt.html>
