# Supplementary material for: Development of the mandibular curve of spee and maxillary compensating curve: A finite element model
Source: PLoS One. 2019 Dec 26;14(12):e0221137. doi: 10.1371/journal.pone.0221137 (PMC6932755; doi:10.1371/journal.pone.0221137)
Supplement: S2 Video — https://www.dentistry.uiowa.edu/orthodontics-curveofspeelongradius-yt.html. (DOCX) [file pone.0221137.s002.docx]

**S2 Video. World Wide Web address of the FEM simulation of human masticatory movements seen in the sagittal plane for C_ROT_ = 400 mm.** [https://www.dentistry.uiowa.edu/orthodontics-curveofspeelongradius-yt.html](https://email.uiowa.edu/owa/redir.aspx?SURL=uz6KILo1mbTHcd577Mw8DR6e7p4jexus0F9P6bJT5YJwr685CajSCGgAdAB0AHAAcwA6AC8ALwB3AHcAdwAuAGQAZQBuAHQAaQBzAHQAcgB5AC4AdQBpAG8AdwBhAC4AZQBkAHUALwBvAHIAdABoAG8AZABvAG4AdABpAGMAcwAtAGMAdQByAHYAZQBvAGYAcwBwAGUAZQBsAG8AbgBnAHIAYQBkAGkAdQBzAC0AeQB0AC4AaAB0AG0AbAA.&URL=https%3a%2f%2fwww.dentistry.uiowa.edu%2forthodontics-curveofspeelongradius-yt.html)
